# Who Are the Key Players Involved with Shaping Public Opinion and Policies on Obesity and Diabetes in New Zealand?

**DOI:** 10.3390/nu10111592

**Published:** 2018-10-30

**Authors:** Willemijn E. de Bruin, Cherie Stayner, Michel de Lange, Rachael W. Taylor

**Affiliations:** 1 Edgar Diabetes and Obesity Research (EDOR) Centre, Department of Medicine, University of Otago, 9016 Dunedin, New Zealand; w.debruin@otago.ac.nz (W.E.d.B.); cherie.stayner@otago.ac.nz (C.S.); 2 Biostatistics Unit, Dunedin School of Medicine, University of Otago, 9016 Dunedin, New Zealand; michel.delange@otago.ac.nz

**Keywords:** social network analysis, diabetes policy, obesity policy, policy network, public opinion, influence

## Abstract

There is an urgent need for strategic approaches to address the high prevalence of obesity and diabetes in New Zealand. Such approaches rely strongly on input from multiple actors in the diabetes and obesity policy space. We conducted a social network analysis to identify influential actors involved with shaping public opinion and/or policy regarding obesity and diabetes in New Zealand. Our analysis revealed a diverse network of 272 individuals deemed influential by their peers. These individuals represented nine professional categories, particularly academics (34%), health service providers (22%), and government representatives (17%). The network included a total of 17 identified decision-makers. Relative capacity of professional categories to access these decision-makers was highest for representatives of the food and beverage industry (25%), compared with nongovernment organisations (9%) or academics (7%). We identified six distinct brokers, in academic (*n* = 4), government (*n* = 1), and nongovernmental (*n* = 1) positions, who could play a key role in improving communication and networking activities among all interest groups. Such actions should ultimately establish effective networks to foster evidence-based policy development to prevent and reduce the burden of diabetes and obesity.

## 1. Introduction

The prevalence of obesity and diabetes is high in New Zealand compared with many developed countries, with New Zealand ranked third for adult obesity prevalence among the Organisation for Economic Co-operation and Development (OECD) countries [1,2]. One in three (32.2%) adults and one in eight (12.3%) children were obese in 2016 [3], and estimates suggest that the average body mass index in New Zealand adults will be more than 30 (the obesity threshold) by the early 2030s [4]. The number of people with diabetes is also of concern; 5.6% of adults have been diagnosed with diabetes, and a further 25.5% are prediabetic [5,6].

The Lancet Obesity Series (2015) highlighted the need for strategic approaches to address the global obesity and diabetes epidemic, including an accountability framework to promote healthy food environments, and to reduce global obesity and associated noncommunicable diseases (NCD) [7,8]. This framework will rely strongly on input from multiple players or actors to meet the agreed objectives and performance outcomes set for obesity and NCD prevention and management. As such, there is a direct need for improved communication and collaboration between all actors [8]. The first step in developing an accountability system, that ultimately includes government, private sector, and civil society groups as well as donors, is to identify all key actors in the field of obesity and diabetes in New Zealand. It is also useful to increase understanding of how these actors interact and to what extent they have power to influence public opinion, policy development, and policy implementation.

A policy network consists of a set of individuals (i.e., actors) with interests in various policy domains and the ability to determine policy success or failure [9]. Aside from politicians and government employees who are involved in policy development, the network can entail a variety of representatives from academic research institutes, the private industry, various interest groups, journalists, and investors. Social network analysis is an established tool to systematically identify, map, and analyse all actors involved with the development and implementation of public policy, as well as advocacy for the policy [10,11,12,13,14,15,16]. This form of social network analysis is also referred to as policy network analysis and has the aim to identify the important actors involved in policymaking and to map their interaction during policymaking processes [17].

Whether and to what extent individual actors or stakeholder groups within a network are able to influence the policy-making process is highly dependent on their ability to access government decision-makers [18,19]. Actors without direct access to a decision-maker communicate via intermediates, who serve as brokers for information exchange within a policy network [19]. Brokers are well-connected individuals who are centrally situated in a policy network and have the potential to bring various subgroups in the network together [20]. Identifying an actor’s position within a network and examining the direct ties of this actor thus allows for an assessment of the overall network structure and identification of powerful and influential positions within this network [10,18].

The aim of this study was to identify a network of (influential) actors involved with shaping public opinion and/or public policies on obesity and diabetes in New Zealand, by performing a systematic social network analysis. Through this network analysis, we aimed to (1) assess how key actors are connected, (2) assess which professional categories these key actors represent, (3) assess to what extent these separate categories interact, and (4) identify actors who hold key positions within the network, enabling them to influence public opinion, policy development, and its implementation.

## 2. Methods

The methodology of this systematic social network analysis is based on an established peer-nomination reputational snowball method [10,12,13,16]. This analytical tool builds on the assumption that (influential) actors within a certain policy domain either know each other in person or by reputation [21,22]. By repeatedly requesting actors to nominate peers whom they consider influential, one can identify sufficient relationships for an accurate description of a policy network.

### 2.1. Data Collection

Snowball sampling commenced with a predefined list of key actors (*n* = 43) representing various professional categories of the obesity and diabetes sector in New Zealand. This so-called seed sample was purposefully selected by seven members of the Edgar Diabetes and Obesity Research (EDOR) centre at the University of Otago (www.otago.ac.nz/diabetes). In order to obtain the seed sample, EDOR members with expertise in the areas of obesity and diabetes, as well as related fields such as public health, Māori and Pacific health, and nutrition, were requested to nominate a seed sample. Consideration was given to the size of the seed sample, inclusion of both obesity and diabetes specialists, as well as ensuring that all professional categories (predefined as: (1) private industry, (2) academics, (3) nongovernment organisations (NGOs) interest groups and professional societies, (4) government, (5) politicians, (6) media, (7) others) were well presented.

The seed sample was contacted by email with the request to nominate (influential) peers involved with shaping public opinion and/or public policies on obesity and diabetes in New Zealand through an online survey (developed with Qualtrics survey software, available in Supplementary 1). In addition, individuals were asked information about the field of expertise of their nominated peers, their connection to these peers, frequency of communication with them, and the peers’ contact information. Participants were also asked to rate their own level of influence on public opinion and public policy regarding diabetes and obesity through four five-point Likert scales [23]. Second-round nominees were subsequently contacted following the same process, thereby commencing the snowball sampling. From the third round onwards, new people were only contacted when they were nominated at least twice. This process was repeated for five rounds, until the network was saturated (i.e., until no new people were nominated at least twice). Reminder emails were sent at two and four weeks after initial invitations, if required. In case participants provided a name of an organisation but nominated no individual, the leader of the organisation (e.g., CEO, executive director, or chairperson) was selected as the representative. Participants were not remunerated for their time.

This study was approved by the University of Otago Human Ethics Committee (reference: 18/042).

### 2.2. Data Analysis

**Figure 1:**
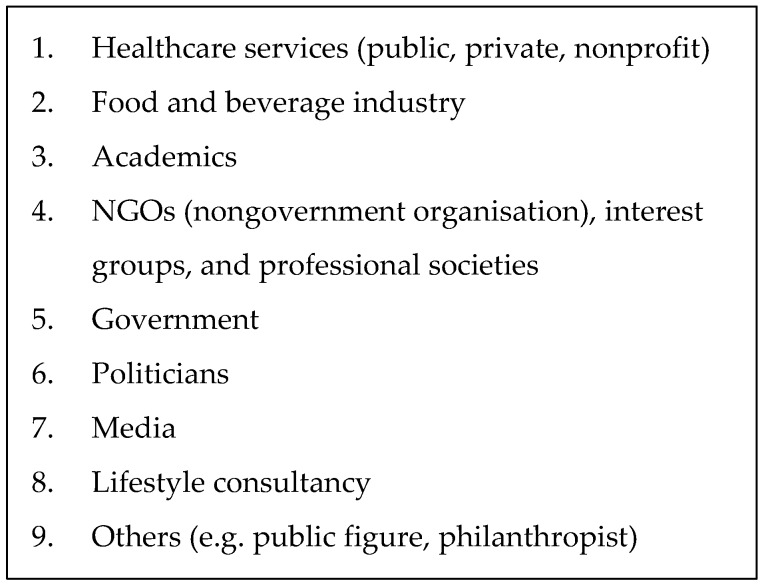
Professional categories for all individuals identified through the network analysis.

All nominees were categorised into professions based on the listed organisation they represented. One category was further defined and two categories were added to the predefined list of professional groups, resulting in nine categories (see Figure 1). ‘Private industry’ was further defined to ‘Food and beverage industry’, all healthcare professionals were merged into the category of ‘Healthcare services’, and ‘Lifestyle consultancy’ emerged as an additional category as these individuals have private businesses but do not fall under the food and beverage industry, registered healthcare services, nor under media (even though in some cases they do have media presence).

Those actors categorised as government representatives or politicians were also categorised as decision-makers or otherwise. In this specific case, decision-maker was defined as an actor who is either a government official or a politician, and who (to some extent) has the power to directly influence public health policy. This includes Ministers, ministerial policy officers, members of parliament, political advisors, and executive leaders of government institutes [10]. This was undertaken through a similar consultative process as completed when establishing the seed sample, and relied on the prior knowledge of four members of the EDOR research team as well as their previous interactions with policy makers in New Zealand.

In some instances (5%), participants did not complete questions detailing whether they were in contact with nominated peers (and the frequency of this contact). In these cases, it was assumed that if a survey respondent nominated someone by name, the two individuals were in direct contact on at least an annual basis (referred to as ‘assumed direct tie’). Respondents who nominated an organisation instead of a person were assumed not to be in direct contact. Missing data (2%) on frequency of contact was entered as ‘annual’ contact for those who indicated to be in direct contact.

**Figure 2:**
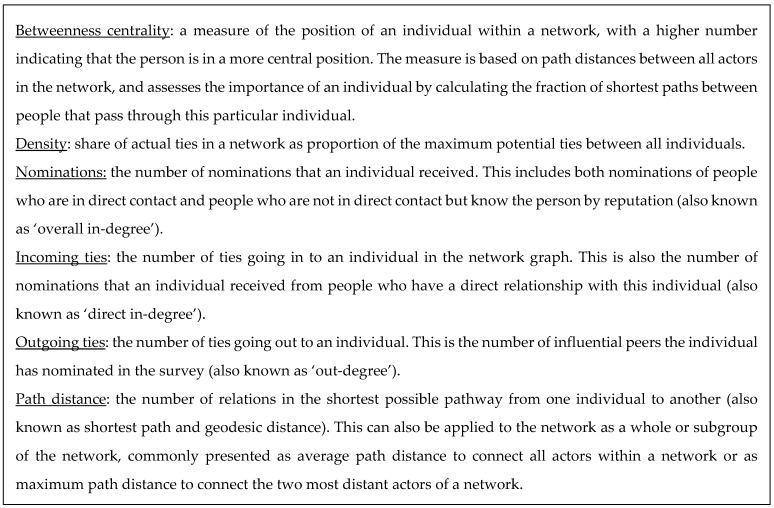
Centrality measures used for this study.

The final datasheet was imported into social network analysis software NodeXL Pro (version 1.0.1.400, Belmont, CA, USA) [24]. Duplicate nominations and self-nominations were deleted. Descriptive statistics and overall network metrics were calculated, including the number of individuals, number of relationships, the graph density, and maximum and average path distances [21,22]. Additional centrality measures were calculated for each individual within the network (see Figure 2 for a comprehensive explanation of each term). We calculated the number of nominations each individual received, the number of direct ties (ingoing and outgoing ties), and the normalised betweenness centrality [17,25].

Network graphs were created using a Harel–Koren fast multiscale algorithm [26]. Direct ties between actors were depicted as a solid connection between nodes, whereas assumed direct ties were depicted as dashed connections. The frequency of direct interaction determined the colour gradient of the ties, ranging from light grey for no contact to dark grey for daily contact. The size of the nodes was determined by the calculated betweenness centrality of each individual, with bigger nodes representing a higher level of betweenness centrality. The professional category of each actor determined the colour and shape of the node in the network (see legend of Figures 4 and 8 in Results section).

Access to decision-makers was assessed by counting all direct relationships with decision-makers within the network. These relationships were subsequently coded as ‘incoming ties’ (i.e., nomination of a decision-maker), ‘outgoing ties’ (i.e., nominations provided by a decision-maker), and ‘relationship between decision-makers’. Relative capacity to access decision-makers was calculated per professional category. Moreover, all path distances between each individual in the network and all identified decision-makers were measured and averaged. Average path distances to decision-makers were also calculated for each professional category.

Cluster analysis was performed to identify subgroups of the network by means of the Clauset–Newman–Moore algorithm, which subdivides all individuals and their relationships into subgroups based on the concept that individuals of a certain cluster have many ties within the group and few with individuals who belong to another cluster [27]. The algorithm is an established functionality of NodeXL to identify clusters of individuals who closely interact with one another.

Regression analyses were performed to assess the relationship between people’s self-rated level of influence and the number of received nominations as well as their calculated betweenness centralities. This was undertaken separately for self-rated influence on public opinion on obesity, on public policy on obesity, on public opinion on diabetes, and on public policy on diabetes (as measured through four five-point Likert scales).

## 3. Results

A total of 188 individuals were invited to participate in the network analysis, of which 131 responded, resulting in an initial response rate of 70%. However, a total of 30 surveys were subsequently excluded, as they were completely empty (*n* = 27) or because respondents completed the survey but were not in direct contact with anyone else in the network (*n* = 3). One hundred and one survey responses were included for data analysis resulting in a final response rate of 54%.

**Figure 3:**
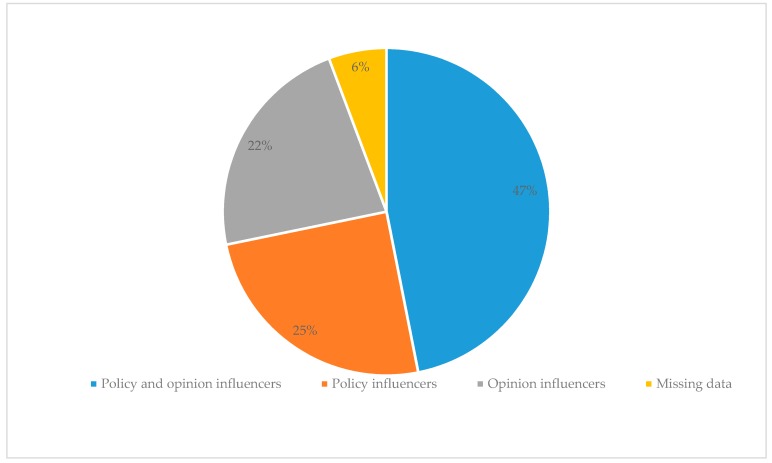
Indicated reason to nominate a peer.

After the removal of duplicate (*n* = 3) and self- (*n* = 2) nominations, the survey provided 804 nominations, including 20 nominations for organisations rather than individuals. Just under half of the nominations (47%) were based on the perceived ability of a peer to influence both public policy and public opinion, 25% of the nominated individuals were regarded as policy influencers, and a further 22% as opinion influencers (Figure 3). The number of received nominations per individual ranged from 1 to 40 nominations for the most frequently nominated actor.

Through five rounds of data collection, we identified a total of 295 unique individuals representing nine professional categories (Figure 1). The response rate was highest among media (83%) and lowest for representatives of NGOs, interest groups, and societies (35%). We received zero responses from the four politicians who were invited for the survey.

### 3.1. The New Zealand Diabetes and Obesity Network

**Figure 4:**
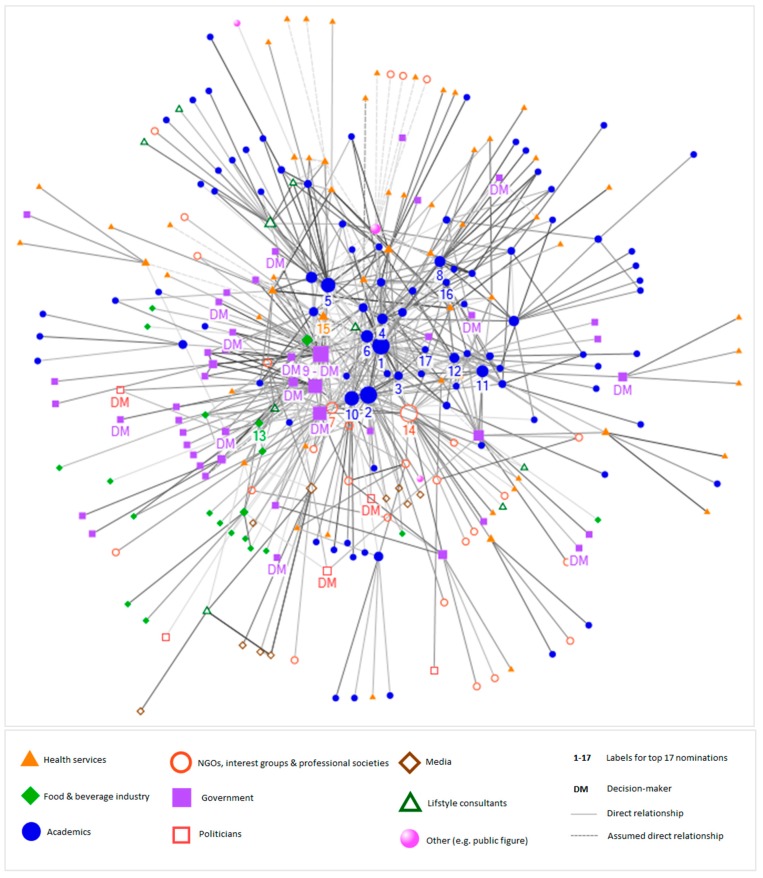
Network graph of overall diabetes and obesity network in New Zealand. The shape and colour of the nodes in the network indicate the professional category of an individual. The size of the nodes represents the betweenness centrality of an individual, with bigger nodes indicating a higher level of betweenness centrality. The grey-scale of the ties between individuals indicates the frequency of communication (ranging from light grey for annual contact to black for daily contact), and dotted lines are assumed direct ties.

Figure 4 is a visual representation of the identified network, depicting people who are in direct contact with one another. The network includes a total of 272 individuals with 659 reported direct ties and a further 38 assumed direct ties (i.e., when information was not provided, ties between individuals were assumed to be direct with annual frequency), depicted as dotted lines. The majority of the reported direct communication with influential peers takes place on a biannual (30%) or monthly (30%) basis.

Individuals who are more centrally situated in the network are predominantly academics and government representatives, whereas healthcare service providers, representatives of NGOs, interest groups and societies, and the media are more peripheral in the network. The network includes a total of 17 identified decision-makers, of which 3 are political decision-makers and 14 are government decision-makers.

The network density is 0.02, which is relatively low and means that only 2% of all potential direct ties (that would connect all individuals in the network with one another) have been identified as actual direct ties in the network. The average path distance is 3.5 with a maximum path distance of 7 between the most distant individuals (see Figure 2 for definition).

**Figure 5:**
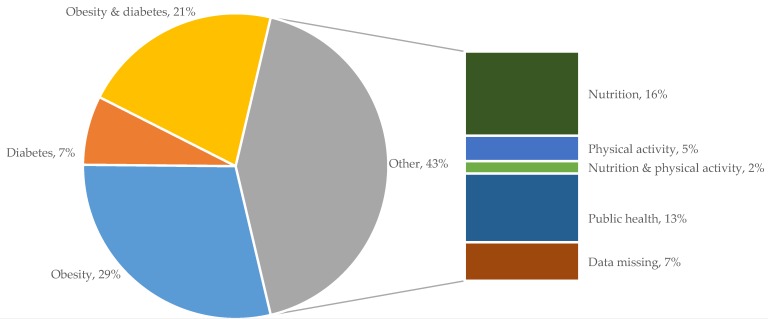
Nominated expertise of individuals in the network. The main pie shows the fraction of key actors who were regarded as obesity and/or diabetes specialists. The grey section groups all individuals with an area of expertise that relates to diabetes and obesity, including nutrition, physical activity, and public health.

More than half of all individuals who are included in the network were regarded as obesity specialists (29%), diabetes specialists (7%), or as individuals who are specialised in both diabetes and obesity (21%). The remaining ties, accumulated in the grey section of Figure 5, were nominations of people considered to be nutrition (16%) or physical activity (5%) specialists, experts in both nutrition and physical activity (2%), or public health specialists (13%).

### 3.2. Interaction between Professional Categories within the Network

The majority of the actors identified through the network analysis are academics (*n* = 93, 34%), health service providers (*n* = 59, 22%), and government representatives (*n* = 45, 17%). Other actors in the network include individuals representing NGOs, interest groups, and societies (*n* = 28, 10%), the food and beverage industry (*n* = 20, 7%), media (*n* = 10, 4%), lifestyle consultants (*n* = 9, 3%), politicians (*n* = 5, 2%), and individuals categorised as others (*n* = 3, 1%). This last group included two philanthropists and one public figure.

Further analysis of the relationships of actors in each professional category revealed that, except for academics, all professional categories reported more ties with people from other professional categories than from their own category (i.e., number and share of external ties exceeded internal ties, Table 1). More than half of the identified relationships of academics were with academic colleagues (51%), while the highest proportion of external relationships were with health services providers (18%).

For most professional categories, the highest number of external ties was to members of academic groups. This is, however, not the case for representatives of the food and beverage industry, with 29% of their ties being with government representatives and a further 25% of their ties as internal connections with colleagues from the same sector. Politicians have the highest share of relationships with government representatives (50%) and representatives of the food and beverage industry (25%).

### 3.3. Access to Decision-Makers and Influential People within the Network

**Figure 6:**
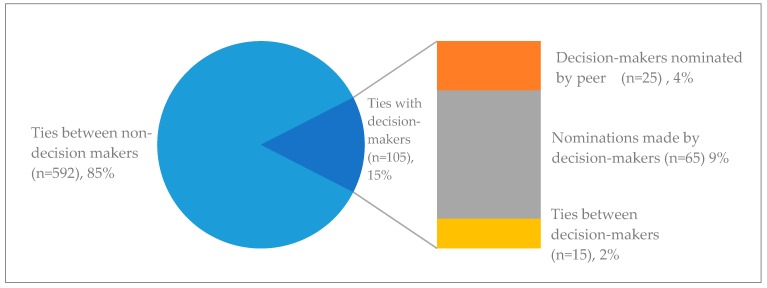
Direct relationships with decision-makers as share of total direct ties in the network. The right bar depicts a further subdivision of type of direct ties with decision-makers (in-degree, out-degree, and tie between decision-makers).

The network entails a total of 697 direct ties, of which 15% are direct ties with decision-makers (*n* = 105). These direct relationships with decision-makers can be further subdivided into those nominated by a peer (also known as incoming-nomination, *n* = 25, 4%), nominations of peers by a decision-maker (also known as outgoing-nomination, *n* = 65, 9%), and relationships between two decision-makers (*n* = 15, 2%). The share of relationships with decision-makers within the identified network is presented in Figure 6 below.

Just over one-third of all 105 identified direct relationships with decision-makers were between decision-makers and academics (36%), followed by non-decision-making governmental representatives (21%) and individuals employed in the food and beverage industry (17%).

**Figure 7:**
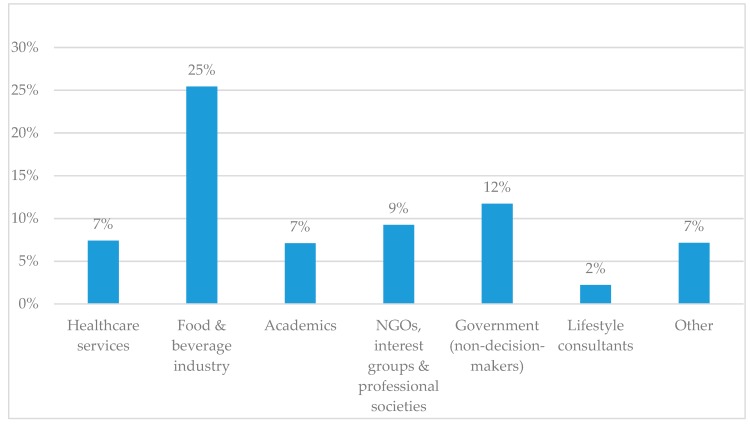
Relative capacity of professional categories to access decision-makers, represented as direct relationships with decision-makers as share of total relationship of the professional category. Media and politicians (non-decision-makers) were left out of the Figure as we found zero direct ties with decision-makers for these categories.

Figure 7 depicts the relative capacity of professional categories to access decision-makers, represented as direct relationships with decision-makers as a share of total relationship within the professional category. A quarter of all identified relationships with representatives of the food and beverage industry is with decision-makers. By contrast, this capacity to access decision-makers is lower for other professional categories, namely 12% for non-decision-making governmental representatives and 9% for representatives of NGOs, interest groups, and professional societies.

Table 2 presents an overview of individuals who received ten or more nominations (range: 10–40). This group includes mostly academics (*n* = 12), two representative of NGOs, one representative of the food and beverage industry, one government decision-maker, and one health service provider. Table 2 also presents the individuals’ path distance to 14 identified decision-makers in this network, and the number of decision-makers to whom they are in direct contact.

The last two columns of Table 2 present the normalised betweenness centrality (NBC) of each individual, and their ranking based on this NBC. As previously addressed, this is a measure of the position of an individual within a network, with a higher NBC indicating that an individual is centrally located in the network and that he/she is often situated on the shortest path connecting all individuals in the network with one another (see Figure 2). In other words, individuals with a high NBC are brokers for information flow within the network. We identified six brokers who had an NBC higher than 0.10 and who also received more than ten nominations. More detailed information on the top six brokers and their position in the network is available in Section 3.4 and Supplementary 2.

We also asked individuals to rate their own level of influence on public opinion and public policy on diabetes and obesity. There were no strong correlations between the self-rated level of influence and the number of received nominations nor the calculated betweenness centralities of people.

### 3.4. Network Clusters

**Figure 8:**
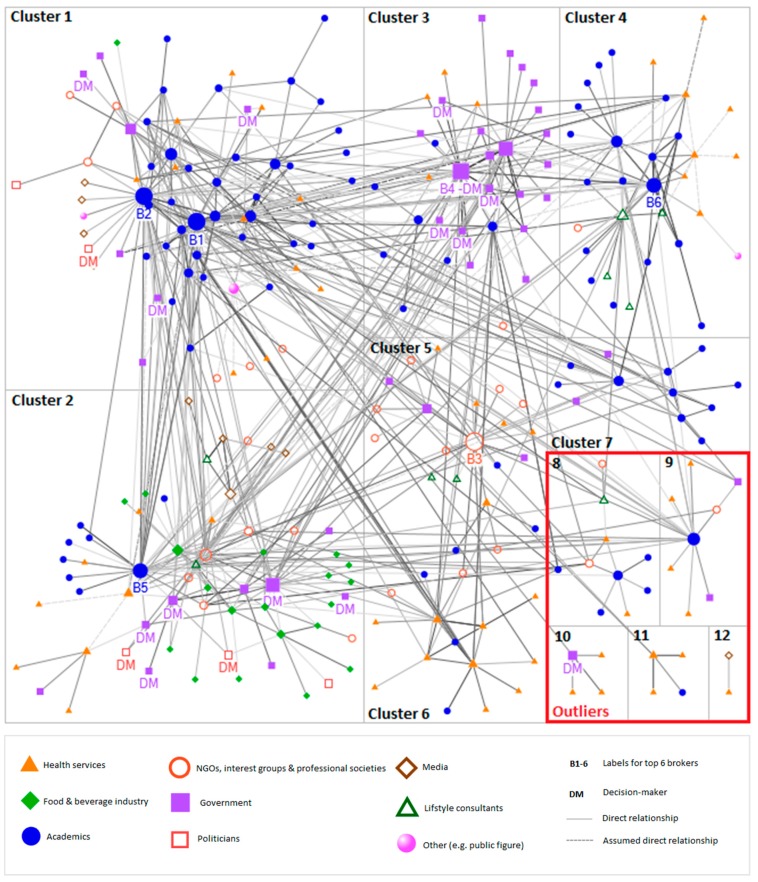
Cluster analysis of the New Zealand Diabetes and Obesity network, distinguishing 12 subgroups of individuals who closely interact with one another.

By means of a cluster analysis of the data set, we identified 12 distinct clusters (Figure 8). These clusters are subgroups of individuals who closely interact with one another. The majority of people in the network were placed in 3 of the 12 clusters.

The biggest cluster, cluster 1, includes 70 individuals, the majority of which were academics (53%). The cluster includes brokers 1 and 2 as well as three government decision-makers and one political decision-maker, who are all in close proximity. The average path distance to all 14 decision-makers in the wider network is 3.1. Other individuals included in cluster 1 are healthcare providers (16%), governmental employees (10%), and representatives of NGOs, interest groups, and professional societies (9%). The cluster consists of 189 direct internal relationships and 124 direct relationships with individuals assigned to other clusters.

The second cluster is more diverse and includes representatives from eight of the nine professional categories. The group consists of 61 individuals with 107 direct internal ties and 87 direct external relationships. This cluster includes all but one representative of the food and beverage industry identified through this network analysis, who jointly account for 31% of the cluster. Other group members are academics (15%), government employees (15%), and health service providers (13%). The greatest number of decision-makers can be found in cluster 2, namely five government decision-makers and two political decision-makers. The average path distance to all 14 decision-makers is 3.1. Cluster 2 includes broker 5.

Cluster 3 includes 33 individuals, who are predominantly government employees (64%) of which 5 were classified as decision-makers including broker 4. Most of the government representatives within this cluster are employed by the Ministry of Health. Other members of the cluster are academics (24%) and health service providers (12%). In contrast to cluster 1 and 2, cluster 3 has more external ties (*n* = 49) than internal ties (*n* = 64), and is in relatively close contact with members of cluster 1 and 2. The average distance to all 14 decision-makers is 2.8.

An overview of group characteristics per cluster is available in Supplementary 2.

## 4. Discussion

Through a systematic social network analysis, we identified a network of 272 individuals deemed influential by their peers. These individuals represented nine professional categories, particularly academics (34%), health service providers (22%), and government representatives (17%). Academics mostly reported direct relationships with fellow academics, while all remaining professional categories reported more relationships with people from other groups. It was noteworthy that food and beverage industry representatives and politicians were the only two categories that did not share a majority of their external ties with academics. The food and beverage industry had most ties with government representatives and with colleagues from the same sector, whereas politicians shared most ties with government representatives and representatives of the food and beverage industry. The network included a total of 17 identified decision-makers. Academics held the highest share of direct relationships with these decision makers (36%) when expressed as a percentage of the total number of direct relationships with decision-makers. However, representatives of the food and beverage industry had greater capacity to access decision-makers, with a quarter of their identified direct relationships being with decision-makers, compared to only 7% for academics. Based on normalised betweenness centrality, we identified six distinct brokers, including four academics, a nutrition specialist employed by an NGO, and a governmental decision-maker specialised in public health.

The size of the network analysed in New Zealand (*n* = 272) is comparable with those mapped through other systematic analyses of policy networks (ranging from *n* = 115 to 390) [11,12,13]. It should, however, be noted that our network includes the policy domains of both diabetes and obesity, as well as influencers of public opinion in addition to policy influencers. Although the size of the network was similar, the density of that network was considerably lower (at 0.02) than what has been observed previously [15,16], implying that the New Zealand network is partially disintegrated. This potential fragmentation is also apparent via the cluster analysis, which revealed 12 distinct clusters in the overall network with clear pockets of academics (cluster 1, 4, and 7), government employees (cluster 3), and representatives of the food and beverage industry (cluster 2). This fragmentation may have occurred because our network analysis covered a wider variety of issues than is typically included, that is, we included both obesity and diabetes (which also yielded many nominees working in the broader nutrition and activity fields), and influence on both public policy and opinion. The various clusters of academics imply limited collaboration between some academic subgroups; again, this may be attributable to the wide variety of policy issues included in this analysis.

Unlike other analyses of policy networks [10,13], we identified academics as the most prominent professional category in the network. Academics also held most direct ties with decision-makers. This implies a potential role for academics to shape public opinion and/or public policies on obesity and diabetes in New Zealand. The ability of health scientists to influence the public policy-making process, in sometimes limited but essential ways, is widely recognised [14,28,29]. Exerting this influence does, however, require active engagement in research–policy relationships, which demands academics to balance traditional, independent, unbiased science with targeted, policy-based research [14,28,29]. It furthermore requires engagement in advocacy, a strategy that some academics are reluctant to employ [14]. On the other hand, previous research suggests that governmental and political decision-makers should also become more aware of the availability of rigorous policy evidence and also require institutional incentive and professional skills to utilise evidence-based analyses and advice in order to establish efficient research–policy relationships [29,30].

Information pathways through which research might influence policy development are fragile and easily interrupted by the political circumstances of governmental agenda setting, commitments, and decision-making [29]. The ability of the food and beverage industry to shape government policy and public opinion in their favour is well known [31]. Although this network analysis does not provide any hard evidence for such disruption, results do indicate that representatives of the food and beverage industry of New Zealand closely interact with decision-makers. Our results show that the food and beverage industry has a high relative capacity to directly access decision-makers, and that their representatives are clustered with decision-makers (cluster 2). These findings are similar to the results of a network analysis of the nutrition policy network in Australia [10]. This potential power and influence of the food and beverage industry should be considered by other stakeholder groups when they attempt to shape public opinion and policies on obesity and diabetes in New Zealand.

The brokers identified through this study hold the potential to steward policy discussions, not only with decision-makers but also within their own personal networks [18,19,20]. Their strategic positions enable them to facilitate opportunities to establish links within the network, and to accumulate and disseminate information [11]. Bridging various, sometimes opposing, coalitions appears to be beneficial in gaining access to governmental decision-makers [18]. The latter suggests that the brokers can play a key role in a networking process, to strengthen the so-called weak ties of the network, in order to ultimately leverage influence of the overall network. Interestingly, we did not find a strong correlation between the identified level of influence and self-rated level of influences, which may indicate that people have limited awareness of their level of influence, perceive power of peers differently than their own level of influence, and/or purposefully under- or overreported their own level of influence. These findings are in contrast to a public health policy network in Australia, which showed that actors were very aware of their own position and level of influence in the network [12].

Although social network analysis is an established tool to unravel policy networks [10,11,12,13,14,15,16], it does rely on the assumption that individuals within a policy domain know each other and can adequately assess each other’s expertise and level of influence [21,22]. Results of this study thus present peer perceptions of influence, which we found to differ from self-rated levels of influence and potentially from reality.

A second limitation of this study relates to its response rate. Individuals who didn’t complete the survey only have incoming nominations and no outgoing nominations, and therefore appeared less connected and more peripheral in the network. Hence, professional categories with a high response rate, such as academics, appear more central and prominent in the network. By contrast, groups with a low response rate, such as representatives of NGOs, interest groups, and professional societies and politicians, were less prominent. Their low response rate may be attributed to a lack of time and political willingness to participate, or both. Academics, on the other hand, were possibly more inclined to complete the survey as the request came from a fellow academic group; this may have led to an overrepresentation of academics in the network. We therefore suggest that future policy network analyses are undertaken by an independent group such as a consultancy company or perhaps by the government itself.

Thirdly, there may have been some response bias. Participants who considered the political implications of the study may have decided to over- or underreport relationships in order to appear more or less influential.

Lastly, we should highlight that data collection and data analysis entailed some decisions. The makeup of the seed sample and the decision to only share the survey with people who were nominated at least twice (from round three onwards) influenced the size and makeup of the network. In addition, we assigned each actor to a professional category whereas some individuals represent more than one in reality (i.e., a medical doctor who is chair of a professional society, or an academic who is board member of an NGO, etc.). This could have been avoided by asking the survey respondents to assign themselves to a professional category they considered most applicable (based on their work hours for various groups); this question was not included in our survey.

Deciding whether someone is a decision-maker or not (binary) was somewhat arbitrary, whereas in reality, public decision-making is a complex process with various tiers of decision-making [32,33]. However, these decisions were made in consultation with at least four EDOR researchers, and in accordance with the methodology of previous social network analyses [10,11,12]. We acknowledge that results of this network analysis could have been validated through interviews with the identified influential actors and decision-makers. This was not feasible due to limited resources but could be explored in future research.

A key strength of this study was the large seed sample, with members chosen across all fields to not bias the results in any direction. Despite the addressed study limitations, we are confident that this study has shed new light on key actors involved with shaping public opinion and policies on obesity and diabetes in New Zealand. New Zealand is a small country in terms of population size, and establishing effective networks to foster evidence-based policy development could thus be easier to accomplish [28,29]. The results of this study can provide direction for the establishment of such a network.

## 5. Conclusions

Through this social network analysis, we identified a diverse network of actors involved with shaping public opinion and public policies on obesity and diabetes in New Zealand. Our results show that the network is partially disintegrated even though it has a manageable size. This implies a need for improved communication and networking activities among all interest groups. The six identified brokers could play a key role in this, as they are in the position to facilitate discussion, improve information flow, and foster collaboration.

## Figures and Tables

**Table 1:** Representation of professional categories within the network and their level of interaction (including number of individuals per professional category, number and share of internal ties and external ties, and communication between professional categories).

Professional Category	Individuals (*n* = 272)	Internal Ties	External Ties	Communication between Professional Categories
	*n* (%)	*n* (%)	*n* (%)	Professional category (%)
**Health services**	59 (22)	31 (19)	**131 (81)**	Academics (50) Health service colleagues (19)
**Food and beverage industry**	20 (7)	15 (25)	**44 (75)**	Government (29) Food and beverage industry colleagues (25)
**Academics**	93 (34)	**230 (51)**	220 (49)	Academic colleagues (51) Health services (18)
**NGOs, interest groups, and societies**	28 (10)	10 (9)	**100 (91)**	Academics (34) Government (23)
**Government**	45 (17)	53 (30)	**124 (70)**	Academics (33) Government colleagues (30)
**Politicians**	5 (2)	0 (0)	**8 (100)**	Government (50) Food and beverage industry (25)
**Media**	10 (4)	6 (30)	**14 (70)**	Media colleagues (30) Academics (25)
**Lifestyle consultants**	9 (3)	4 (9)	**41 (91)**	Academics 47) Food and beverage industry (13)
**Other**	3 (1)	0 (0)	**14 (100)**	Academics (50) Health services; NGOs, interest groups and societies (21)

NGOs, nongovernment organisations.

**Table 2:** Influential people and brokers ranked by number of votes received, presented centrality measured includes average path distance to decision-makers, direct access to decision-makers, and normalised betweenness centrality.

Expertise and Professional Category	Nominations	Average Path Distance to All DMs	Direct Access to DMs	Centrality (NBC)	Ranking Based on Centrality
**Obesity Specialist, Academic**	**40**	**1.8**	**4**	**0.17**	**1 ^b^**
**Diabetes and Nutrition Specialist, Academic**	**26**	**1.9**	**4**	**0.17**	**2 ^b^**
Nutrition Specialist, Academic	22	2.6	0	0.02	36
Obesity Specialist, Academic	20	2.2	1	0.04	16
**Physical Activity Specialist, Academic**	**18**	**2.2**	**3**	**0.11**	**6 ^b^**
Obesity and Nutrition, Academic	17	2.3	1	0.07	9
Nutrition Specialist, NGO	14	2.2	2	0.05	15
Obesity Specialist, Academic	13	2.6	0	0.05	13
**Public Health Specialist, Government (DM)**	**12**	**1.7 ^a^**	**6 ^a^**	**0.13**	**4 ^b^**
**Nutrition Specialist, Academic**	**12**	**2.1**	**3**	**0.11**	**5 ^b^**
Diabetes Specialist, Academic	12	2.4	1	0.07	10
Obesity Specialist, Academic	12	2.7	0	0.04	22
Nutrition Specialist, Food and Beverage Industry	12	2.1	3	0.02	37
**Nutrition Specialist, NGO**	**11**	**2.3**	**2**	**0.15**	**3 ^b^**
Nutrition Specialist, Health professional	11	2.5	1	0.04	21
Diabetes Specialist, Academic	11	2.9	0	0.01	70
Obesity Specialist, Academic	10	2.7	0	0.00	87

DM(s), decision-maker(s); NBC, Normalised Betweenness Centrality, NGO, nongovernment organisation. ^a^ This individual is a governmental decision-maker, the presented average and count only include access to other DMs. ^b^ These people are the top six brokers in this network.

## Supplementary Materials

The following are available online at http://www.mdpi.com/2072-6643/10/11/1592/s1.
