# Supplementary material for: Who Are the Key Players Involved with Shaping Public Opinion and Policies on Obesity and Diabetes in New Zealand?
Source: Nutrients. 2018 Oct 30;10(11):1592. doi: 10.3390/nu10111592 (PMC6267561; doi:10.3390/nu10111592)
Supplement: Supplementary file 1 [file nutrients-10-01592-s001.pdf]

Supplementary 1: Questionnaire

Study participants were contacted by email with the request to nominate (influential) peers involved with shaping public opinion and/or public policies on obesity and diabetes in New Zealand through the following survey (developed and distributed with Qualtrics online survey software.):

The Edgar Diabetes and Obesity Research Centre is undertaking a network analysis to identify key people involved with shaping public opinion and/or public policies on obesity and diabetes in New Zealand. Given that you have been identified as a key person, we would like to invite you to nominate other people who you consider important in the New Zealand obesity and diabetes sector. People to be considered for such nomination, would in some capacity be able to:

- influence public opinion,
- undertake research with the potential to inform public opinion,
- place a topic on the public agenda,
- undertake research with the potential to inform public policy,
- place a topic on the policy agenda,
- shape public policy ideas,
- initiate public policy proposal,
- substantially change or veto public policy proposals, and/or
- substantially affect public policy implementation.

You are invited to nominate as many or as few people that you can think of (up to one hundred responses are possible). If you cannot identify an individual, please provide the name of any organisation(s) that you feel meets these criteria.

	**Please provide full name and affiliated organisation(s) of nominated person:**		**What is his or her area of expertise? (tick the box, multiple answers possible)**				**He or she can influence: (tick the box, multiple answers possible)**		**Are you in direct contact with this person? (one answer)**	**How often are you in direct contact? (one answer)**	**Please provide email address, if available:**
	Name:	Organisation:	Obesity	Diabetes	Nutrition	Physical Activity	Public opinion	Public policy	*Yes, No.*	*Daily, Weekly, Monthly, Bi-annually, Annually.*	
**1**											

**2**											
**3***											

* The survey had no required minimum number of peer nominations in order to avoid that additional actors with less influence would be added to the list, and allowed a maximum of 100 nominations.

Lastly, we would like to know how you would rate your own capacity to influence public opinion and public policies on obesity and diabetes in New Zealand, based on the following 5-point scale.

	**1 - not at all influential**	**2 - slightly influential**	**3 - somewhat influential**	**4 - very influential**	**5 - extremely influential**
**Public opinion on obesity**					
**Public policy on obesity**					
**Public opinion on diabetes**					
**Public policy on diabetes**					

Supplementary 2: Overview of group characteristics per cluster

Cluster	Description	Individuals (n = 272)	Internal ties (%)	External ties (%)	Maximum Path Distance	Average Path Distance	Density	DMs within group	Average distance to DMs
**1**	Pre-dominantly academics – including Broker 1, Broker 2 and three GDMs and one PDM.	70	189 (60)	124 (40)	6	2.8	0.07	4	3.1
**2**	Diverse cluster, including all but one representative of the food and beverage industry. Highest number of DMs (5 GDMs and 4 PDMs). Broker 5.	61	107 (55)	87 (45)	6	3.1	0.06	7	3.1
**3**	Majority government officials, of which 5 are GDMs.	33	49 (43)	64 (57)	4	2.4	0.09	5	2.8
**4**	Cluster of academics including Broker 6, healthcare providers, and lifestyle consultants. No DMs included.	32	63 (60)	42 (40)	4	2.5	0.11	0	3.5
**5**	Cluster with highest share of representative of NGOs, interest groups and professional societies, including Broker 3.	21	21 (48)	23 (52)	4	2.3	0.10	0	3.5
**6**	Cluster of health service providers	15	20 (47)	23 (53)	3	2.0	0.17	0	3.6
**7**	Small cluster of mainly academics	12	13 (38)	21 (62)	4	2.1	0.20	0	3.5
**8**	Outliers	10	10 (42)	14 (58)	4	2.0	0.22	0	3.5

**9**	8	7 (25)	21 (75)	3	1.7	0.25	0	3.0
**10**	4	3 (60)	1 (40)	2	1.1	0.50	1	4.0
**11**	4	3 (75)	2 (25)	2	1.1	0.50	0	4.0
**12**	2	1 (100)	0 (0)	1	0.5	1.00	0	N/A

DM stands for decision-maker, PDM for political decision-maker, and GDM for governmental decision-maker. Internal ties are all incoming and outgoing ties with people in another cluster, whereas external ties are all relationships within the cluster. Maximum path distance is the length of the path between the most distant individuals within a cluster, and average path distance is the average number of steps to connect all individuals from a cluster.
